# The Retinal Symptoms of Vascular Degeneration

**Published:** 1905-11-18

**Authors:** 


### THE RETINAL SYMPTOMS OF VASCULAR DEGENERATION.

Dr. L. A. W. Alliman[1] believes that in some cases it is possible by an inspection of the fundus oculi to recognise those functional changes in the blood-vessels which precede organic degeneration. Hence, as the condition of the retinal arteries is doubtless an index of the state of the arterial system generally, degenerative tendencies may be detected in their earliest developments and the patient may thus be guided and protected before irreparable changes have made repair impossible. After examination of a large number of patients, including a study of the retinal vessels and a survey of the general physical condition, he divides his cases into four groups, recognising at the same time that these are not separated from one another by hard and fast lines, but shade insensibly one into the other. In the first group the important finding is a tortuosity of the smaller retinal vessels and of their terminal twigs; this is seen as a "crinkling and sharp turning of the vessel upon itself" as distinct from the sinuous curve of the vessels seen in neuro-retinitis and other conditions. With this twisting there may be a more or less general haze, probably due to œdema of the retina. In the second group, along with the appearances just described, there is also seen at the point where one vessel crosses another a curving of the more superficial vessel, as if it rose from its level to bend over the deeper one. Together with this, in many cases, is observed a change in the calibre of the retinal vessels in the form of temporary contractions seen in small areas and due to spasm. Another feature seen in this group is momentary decrease in the force of the retinal circulation. This is appreciated in the form of a "flash," difficult of exact description, but giving the impression that something has happened, the nature of which one is unable to determine. Another indication of lack of force in the circulation is found in the ease with which the retinal circulation is impeded by slight pressure with the finger on the eyeball. In the third group of cases there is evidence of organic change in the vessel walls. The tortuosity above described may or may not be present, but in any event it has probably preceded the changes now to be described. At the crossings the curve of the more superficial vessels is pronounced and the blood-stream of the underlying vessel is somewhat obscured by the wall of the vessel which covers it. In this latter there may be seen on each border of the blood-stream a slight haze as if from some early thickening of the arterial coats. Spasmodic changes in calibre are not usual. Sometimes minute hæmorrhages or white areas left by their absorption may be seen. In the fourth class are included cases presenting advanced vascular disease, such as is met with in hæmorrhagic, albuminuric, and diabetic retinitis. Here, in most instances, the diseased condition of the vessels is seen in deep depressions at the line of crossing, in interference with the blood flow in the deeper vessel, and as prominent white lines bounding the course of the superior vessel.

The slighter and earlier retinal changes are of special importance, as they indicate a high peripheral tension which interferes with proper tissue metabolism. The patients may be free from all signs of organic disease and often believe themselves to be in good health, though a complaint of lassitude or lack of energy is not uncommon. The one constant abnormality is some disturbance of elimination as can be demonstrated by a diminution in the excretion of urea. Thus a study of the retinal vessels may give an early indication of those functional vascular changes which are probably the primary movement in a development that leads, if not checked, to gross organic disease.

[1] Amer. Med. Vol. VII., No. 8.

